# Hepatitis C Infection Associated with Acquired Pure Red Cell Aplasia

**DOI:** 10.3390/tropicalmed8010008

**Published:** 2022-12-22

**Authors:** Destini Teague, Carmelo Gurnari, Hussein Awada, Jaroslaw P. Maciejewski, Ibrahim Ibrahim, Taha Bat

**Affiliations:** 1 Division of Hematology-Oncology, UT Southwestern Medical Center, Dallas, TX 75390, USA; 2 Translational Hematology and Oncology Research, Cleveland Clinic, Cleveland, OH 44195, USA; 3 Department of Biomedicine and Prevention, University of Rome Tor Vergata, 00133 Rome, Italy

**Keywords:** pure red cell aplasia, HCV, bone marrow failure syndromes

## Abstract

Acquired pure red cell aplasia is a rare bone marrow failure disorder characterized by many underlying etiologies. The hallmark bone marrow feature is the near absence of erythroid precursors that otherwise exhibit normal cellularity, which has been attributed to both immune- and cellular-mediated mechanisms. Besides being merely speculative and considering the rarity of the disorder, the description of acquired pure red cell aplasia clinical associations represents a unique occasion to improve our current clinical knowledge of the disease, reveal clues on its pathogenesis, and guide therapeutic decisions. The varied clinical scenarios and common acquired pure red cell aplasia associated conditions (i.e., thymoma, T cell/NK-cell large granular lymphocyte leukemia, B cell dyscrasia) suggest a heterogeneity of pathogenic routes. Viral etiologies must always be considered and worked up in the initial assessment of newly diagnosed acquired pure red cell aplasia patients. In this report, we present two cases of hepatitis-C-related acquired pure red cell aplasia and successful use of anti-viral strategies in the achievement of a complete response.

Pure red cell aplasia (PRCA) is a rare hematological disorder defined by normocytic anemia, reticulocytopenia, and absence of erythroblasts in the bone marrow (BM) [1]. Acquired cases in adults must be differentiated from inherited disorders, namely Diamond-Blackfan anemia, typically presenting in early childhood and caused by mutations of ribosome biogenesis genes [2]. Some known etiologies of acquired PRCA include autoimmune disorders, lymphoproliferative neoplasms (e.g., T-cell large granular lymphocyte leukemia, T-LGLL), solid tumors (chiefly thymoma), and infections mostly in the context of immunodeficiencies (e.g., B19 parvovirus-mediated transient aplastic crisis) [1,3,4,5]. However, the majority of cases are supposed to be idiopathic or somehow linked to cytotoxic T-cell proliferations [3].

Being a multifaceted disorder, the precise understanding of the underlying pathogenic mechanism represents the key for a rational therapeutic approach [6]. For instance, surgery is indicated in thymoma-associated cases, whereas the response to immunoglobulin supplementation (IVIG) in cases negative for B19 parvovirus may point towards the pathogenic role of other viral infections [7]. Herein, we present two cases of newly diagnosed acquired PRCA associated with concomitant hepatitis C virus (HCV) infection.

The records of patients diagnosed with PRCA at the University of Texas Southwestern and Cleveland Clinic Foundation between 2000 and 2022 were reviewed for the presence of hepatitis C infection. Clinical, laboratory, and molecular data were abstracted in accordance with guidelines set forth by the Declaration of Helsinki and the participating institutions. Among a total of 81 patients, 2 were identified as acquired PRCA cases potentially associated with hepatitis C after ruling out other conditions as per established guidelines [7].

Patient #1 was a 44-year-old African American woman who presented with hematochezia, hematuria, fatigue, generalized weakness, and headache. A complete blood count (CBC) revealed anemia, and peripheral blood smear was significant for anisocytosis and poikilocytosis, with presence of ovalocytes, spherocytes, and echinocytes, rare bite cells and few schistocytes (Table 1). During work-up for underlying causes of anemia, she tested positive for hepatitis C infection (genotype 1a/b) with a viral load of 630,000 IU/mL (HCV RNA PCR), whereas other viral serologies, micronutrients, and iron deficiencies as well as metabolic disorders were ruled out. A diagnosis of PRCA was suspected and confirmed via BM evaluation, which revealed 50–60% cellularity with marked erythroid hypoplasia, no dysplastic changes, and normal cytogenetics. Despite no evidence of liver alterations at ultrasound and at a CT of the abdomen, a decision to proceed to a liver biopsy was made, and fibrosis Metavir classification was F1 [8]. Treatment included transfusion support with six units of red blood cells (RBC) during hospital admission followed by eight weeks of outpatient treatment with daily sofosbuvir/velpatasvir 400/100 mg. A most recent follow up 8 months after initial PRCA diagnosis showed sustained virological response, no evidence of cirrhosis on ultrasound elastography, and normalized blood counts at CBC (last hemoglobin levels were 12 g/dL).

Patient #2 was a 58-year-old African American woman with a history of advanced HIV with suboptimal compliance to highly active antiretroviral therapy (HAART) and concomitant HCV infection (prior to the era of anti-HCV direct-acting antivirals, DAAs), who presented with fatigue and dyspnea on exertion. She was found to have normocytic reticulocytopenic anemia and neutropenia with anisocytosis, occasional tear drop, and target cells on peripheral blood smear (Table 1). After appropriate laboratory assessment and imaging, a diagnosis of PRCA was confirmed via BM biopsy, which showed 50% cellularity and early arrest in erythroid development with no signs of dysplasia nor cytogenetic abnormalities. A B-19 parvovirus PCR was negative. Treatment included three units of RBC and IVIG (0.4 g/kg) over four days for two cycles with prompt hematologic response (hemoglobin 13.7 gr/dL) noted after the second course. Nonetheless, the patient discontinued the hematology follow-up after achieving complete remission. Soon thereafter, she developed AIDS after voluntarily stopping HAART and died 32 months following the PRCA diagnosis.

Given the lack of clinical trials, which are difficult to envision in such a rare condition, the medical knowledge on management of PRCA relies on the growing evidence derived from reports of retrospective case series. Therefore, we here describe the unusual association of PRCA and hepatitis C infection. Severe autoimmune cytopenias have been previously observed in treatment-naive HCV positive patients in the pre-DAAs era [9]. Furthermore, case series have also reported PRCA in the setting of hepatitis A and B [10,11]. While in patient #1 the etiology of PRCA may be most certainly ascribed to the HCV infection, in the second case the co-infection with HIV might also be an important determinant. Indeed, in a study on treatment-naive HCV cases also presenting a meta-analysis in the literature, an HIV co-diagnosis was noted in 10% of patients presenting autoimmune cytopenias [9]. The response to DAAs in the first case and to IVIG in the second one points toward the possibility of a viral etiology involved in the pathogenic process triggering PRCA. In such scenario, virus-mediated direct cytotoxic effects, or phenomena of molecular mimicry against erythroid precursors may be invoked [1,3]. 

In summary, we here report on two cases of a rare presentation of acquired PRCA with concurrent hepatitis C infection, which resolved with adequate anti-viral strategies. A precise determination of PRCA etiology by means of an extended work-up is essential to establish a rational therapeutic management. 

## Declarations

All procedures were carried out in accordance with guidelines set forth by the Declaration of Helsinki.

## Figures and Tables

**Table 1:** Clinical Characteristics at onset and follow-up of patients with hepatitis C and pure red cell aplasia.

Variable	Patient 1	Patient 2
Age (years)	44	58
Sex	F	F
Race	African American	African American
Symptoms at Presentation	hematochezia, hematuria, fatigue, generalized weakness, headache	fatigue, dyspnea on exertion
Hepatitis Testing Method and Results	HCV RNA PCR +, HCV quant 630,000, HCV genotype 1a or 1b	Serology
Hepatitis Severity	ALT 34, AST 54, GGT 38, fibrosis metavir classification F1; CT Abdomen Pelvis w/IV contrast liver unremarkable, portal and hepatic veins patent; US unremarkable	ALT 63, AST 124, CT Abdomen Pelvis w/IV contrast liver unremarkable, portal and hepatic veins patent; RUQ US unremarkable
Timing	Concomitant	Previously known HCV/HIV positivity
WBC, ×10^9^/L	4.34	1.42
Hemoglobin, g/dL	6.2	5.4
MCV (fL)	86.4	83.6
Platelets, ×10^9^/L	154	181
ANC	1.98	0.54
Reticulocytes	0.2%	0.3%
DAT	negative	negative
Bone Marrow Biopsy Results	50–60% cellularity, erythroid hypoplasia; no increase in blasts (1–2%); heterogeneous interstitial and paratrabecular lymphoid aggregates and histiocytes	50% cellularity, early arrest in erythroid development
T-cell rearrangement	Negative	Not performed
Parvovirus PCR/ELISA	Both IgG and IgM negative	PCR negative
Cytogenetics	46,XY [20]	46,XY [20]
Treatment	sofosbuvir/velpatasvir 400–100 mg daily ×8 weeks	IVIG (0.4 g/kg) ×2 cycles of 4 days each
Most recent follow up (time since diagnosis)	8 months	32 months
Hematology Treatment Response	Complete response	Complete response
Hepatitis Treatment Response	SVR12 (sustained virological response determined by HCV viral load not detected at least 12 weeks after completion of therapy); no evidence of cirrhosis per US elastography	Not available, patient discontinued hematology follow-up after achieving complete remission and soon thereafter developed AIDS after voluntarily stopping HAART

F: female; PCR: polymerase chain reaction; HCV: hepatitis C virus; ALT: alanine transaminase; AST: aspartate aminotransferase; GGT: gamma-glutamyl transferase; RUQ: right upper quadrant; US: ultrasound; CT: computed tomography; WBC: white blood cells; MCV: mean corpuscular volume; ANC: absolute neutrophil count; ELISA: enzyme-linked immunosorbent assay; HAART: highly active antiretroviral therapy. CBC counts are given at PRCA onset.

## Data Availability

Not applicable.
